# Fears and misconceptions toward COVID‐19 vaccination among Syrian population: A cross‐sectional study

**DOI:** 10.1002/hsr2.1426

**Published:** 2023-07-12

**Authors:** Mohamad Klib, Munir Ghandour, Osama Alazki, Ayman I. Nabhan, Fatima A. Idres, Homam Alolabi, Majd S. Khaddour, Jaafar Zahlout, Farah Albakkar, Hasan M. M. Hamoud, Hasan N. Al Houri, Bana Z. Alafandi, Data Collection Group

**Affiliations:** ^1^ Faculty of Medicine Damascus University Damascus Syria; ^2^ Internal Medicine Department Damascus University Damascus Syria; ^3^ Faculty of Medicine Tishreen University Latakia Syria; ^4^ Faculty of Medicine Al Andalus University for Medical Sciences Tartus Syria; ^5^ Faculty of Medicine AL‐Baath University Homs Syria; ^6^ Faculty of Medicine Syrian Private University Damascus Syria; ^7^ Faculty of Medicine University of Aleppo Aleppo Syria; ^8^ Faculty of Dentistry University of Kalamoon Deir Atieh Syria

**Keywords:** COVID‐19, effectiveness, fear, side effect, Syria, vaccine, vaccine acceptance

## Abstract

**Background and Aims:**

Despite the significant milestone of vaccine discovery, the spread of misinformation and pseudoscientific claims has resulted in an increasing number of people refusing vaccination in Syria. In this study, we aimed to explore fears and misconceptions towards COVID‐19 vaccines among the Syrian population.

**Methods:**

We conducted a nationwide cross‐sectional study between January and May 2022, using a convenience sample of 10,006 participants aged at least 18 years and living in Syria. We administered a validated online/paper questionnaire and conducted face‐to‐face interviews. We used SPSS software (version 26) for statistical analysis, assessing our data using frequency and *χ* ^2^ tests, with *p* < 0.05 considered statistically significant.

**Results:**

The majority of the participants were female 6048 (60.4%), university degree holders 7304 (73%), and from urban areas 8015 (80.1%). Approximately half of the participants 5021 (50.2%) belonged to the medical sector (49% had concerns about the vaccine). Females, university degree holders, and participants with a history of symptomatic COVID‐19 were more likely to have fears about the vaccines. The main concerns about the vaccines were the rapid development, fears of blood clots, and common side effects. The prevalence of some misconceptions was relatively high, such as the belief that the vaccine is an experiment or a secret plan to reduce the population. Reliable sources are crucial to fight misleading information on social media.

**Conclusion:**

COVID‐19 vaccine is key to controlling the spread, but acceptance rate is critical. High variability in vaccine acceptance and high vaccine hesitancy can affect the efforts to terminate the COVID‐19 pandemic. Addressing the barriers associated with the acceptance of COVID‐19 vaccination will be the cornerstone to achieving maximum vaccination coverage. It is important to consider the reasons for refusing the COVID‐19 vaccine when interpreting the results of any study on vaccine attitudes among the Syrian population.

## 1 INTRODUCTION

The COVID‐19 pandemic has revealed weaknesses in health systems worldwide, which can affect confidence in government, economy, and social unity. It has yielded many implications for health systems such as insurance coverage, financial losses for providers, substantial racial and ethnic disparities, and crises in public health.^1^ As of the November 4, 2022, there have been 628,694,934 confirmed cases of COVID‐19 Globally, including 6,576,088 deaths, reported to the world health organization (WHO). In the Syrian Arab Republic, from January 2020 to April 2023, 57,374 confirmed cases of COVID‐19 with 3163 deaths.^2^

Although vaccine discovery was one of humanity's greatest milestones, we stand today against pseudoscience and misinformation's plead for irrational side effects and its ineffectiveness against diseases, which in turn has led many children and adults not to take their required vaccines, these concerns are rising even in the vaccinated.^3^, ^4^ Long before the SARS‐COV2 pandemic, the number of people refusing vaccination was increasing and although vaccines like any other medical drug are associated with several side effects, they are considered a normal reaction of the vaccines and not necessarily a negative reason to halt its administration, except for specific cases with known allergies to vaccine's components or have impaired immunity. Vaccine acceptance could be improved through social guidance campaigns as humanity is currently going through an “infodemic” which may be responsible for increased morbidity and mortality of several infectious diseases. Social media has been responsible for aggravating many existing fears of vaccines and helped “anti‐vax” campaigns reach out to a large percentage of the population and provide convincing claims against vaccines and their efficacy.^4^, ^5^

Since the first COVID‐19 vaccine by Pfizer dose that was registered in December 2020, the first dose of the COVID‐19 vaccine was not admitted in Syria until the first of April 2021, and as of November 10, 2022, a total of 4,777,586 vaccine doses have been administered which accounts for only 13% of the total population and only 2,105,113 persons were fully vaccinated in Syria, whereas a total of 12,861,382,558 vaccine doses worldwide have been administered.^2^

With the new wave of information that hit social platforms since the announcement of the first vaccine, different opinions clashed, and there was no clear consensus on what the majority wanted. Yasmin and colleagues found that 87.8% of the study cohort indicated a willingness to get vaccinated, with certain factors, such as younger age and lower attained education, being associated independently with vaccination hesitancy.^6^ In Japan, Machida et al.^7^ have found that individuals with any social capital are more likely to receive a COVID‐19 vaccination than those with none, suggesting that social capital may be a factor that can reduce vaccine hesitancy during a pandemic. Gautier et al.^8^ have found high rates of vaccine hesitation in health sciences students, which in turn may raise concerns about vaccine acceptance among healthcare practitioners and its influence on the population's acceptance.

In Syria, Shibani et al.^9^ found that both fears of side effects and mistrust in the rapid development of vaccines would steer the efforts away from eliminating the pandemic. However, at the same time of the study, new campaigns were launched to raise awareness in the population and the results of these campaigns were yet to be discovered. In addition, The Shibani and colleagues study's objectives were regarding vaccines in general. Since a lot of vaccines with different mechanisms of action were released to the public afterwards, there was an increasing need to know which type of vaccine Syrians would prefer and how this would later affect vaccination rates. Swed et al.^10^ have suggested that in addition to the aforementioned factors rural areas may have the highest rate of vaccine hesitancy due to factors like high rates of poverty and poor infrastructure. To our knowledge, no study was conducted to investigate the vaccination rates in these areas.

According to Mohammad et al.^11^ the reasons for this low rate of vaccination are delayed vaccine availability in Syria and low acceptance of COVID‐19 vaccines among the Syrian population.

The objective of this nationwide study is to provide insights into the factors that contribute to vaccine hesitancy, identify the most common fears and misconceptions, and inform public health strategies to improve vaccine uptake and address vaccine‐related concerns in Syria.

## 2 MATERIALS AND METHODS

### 2.1 Study design, sampling technique, and data collection procedures

We conducted a nation‐wide cross‐sectional study by circulating an online/paper questionnaire and a face‐to‐face interview between January 30 and May 1, 2022. The target population was the adult general people of all governorates in Syria (Damascus, Rif Dimashq, Aleppo, Homs, Hama, Idlib, Lattakia, Tartous, Deir ez‐Zor, Daraa). The calculated sample size was 9599 with an expected acceptance prevalence of 50%, a confidence level of 95%, an acceptable margin of error of 1%, and considering the population of Syria in 2022 is 18,563,379 people.^12^ The sample size was calculated with https://www.checkmarket.com/sample-size-calculator/. The inclusion criteria were individuals who were at least 18‐year‐old, voluntarily agreed to participate in this study, and living in Syria. Paper questionnaire and a face‐to‐face interview aimed to reduce the sampling error, increase the study power, and ensure that the questionnaire will reach all individuals such as the elderly, people with no internet connection, and people who do not have mobile phones and social media.

The questionnaire was created by the authors after an extensive review of the literature, designed via Google form, and disseminated the electronic link through social media networks (Facebook, WhatsApp, Telegram, Instagram, and Twitter). The paper questionnaire was distributed in all suitable places (hospitals, private clinics, pharmacies, markets, and university housing).

The data was collected by 58 collaborators from different cities in Syria, each collaborator had to collect approximately 150–200 answers. Thirty collaborators collected data through an online questionnaire with 20 face‐to‐face interviews. Seventeen collaborators collected data through both electronic and paper questionnaires. Ten collaborators collected data through only a printed questionnaire. The collaborators were trained to conduct the data collection process to ensure accuracy and validity. To ensure the accuracy and validity of the data collection process, the first author trained each of the collaborators on the study objectives and procedures, reviewed the questionnaire and its variables with them, and provided guidance on how to approach participants and collect the data. The collaborators were also trained on how to handle illiterate participants and avoid duplication of participants by asking if they had already participated in the survey.

To collect the data, a set of questions were constructed. The questionnaire was first pre‐tested, revised, and finalized based on a pilot sample (*n* = 30) to assess the questionnaire's clarity. All questions were simple, and clear with multiple response options and were written in the Arabic language as it is the native language of the Syrian population. Informed consent included “Yes or No” question was obtained at the beginning of the questionnaire to ensure the approval of participant, the survey was voluntary and anonymous, response to all questions was not mandatory and and participants were allowed to withdraw from the survey at any time. No personal identifications were obtained, and all data maintained confidential without posing any threat according to the Syrian culture and traditions. Time for the questionnaire completion was approximately 12 ± 5 min.

To make sure that our sample is representative and not biased, we used multiple strategies to reach a diverse group of participants. We used a probability‐based approach to select participants from both urban and rural areas across various governorates. Additionally, we used a convenient sampling method to reach individuals who may be harder to access through traditional means, such as the elderly or disabled. We also utilized the snowball sampling method to increase the size and diversity of our sample.

### 2.2 Structure and content of the questionnaire

There were 22 questions in the questionnaire and they were categorized into five sections:

1. The first section requested participants to complete 12 questions about their social demographic characteristics age, gender, place of residence (urban, rural, or outside the country), the governorate, education level, studying or working in healthcare, health insurance, the amount of risk that COVID‐19 poses toward the community and the participant in person and if they had any previous COVID‐19 infection or any family member had died from COVID‐19.
2. The second section consisted of five questions to gather information about preferable vaccines and if they had received the vaccine. In case the person did take the vaccine, he will be asked further questions and will be moved directly to the fourth section but if the person did not take the vaccine, he will be moved directly to the third section
3. The third section asked one question about the willingness of participants to receive the COVID‐19 vaccine in the future.
4. The fourth section included about the hesitancy and refusal to take the vaccine, in case the person answered No, the questionnaire will end directly, but if the person had fears about the vaccine he will be moved directly to the final section. We divided the study sample into two groups according to this question.
5. The final section assessed the fears toward enforcing vaccination through a 5 point‐Likert scale (from 1 to 5‐extreme fear of imposing the vaccine), in addition to the reasons that prevent the participant from receiving the vaccine which divided into five sections (side effects, effectiveness, general fears, manufacturing, conspiracy theories).

Quantitative variables were collected based on subgroups. Age was divided into four groups (18–24/24–44/4–65/>65), and attitudes toward enforcing vaccination were assessed using a 5‐point Likert scale.

### 2.3 Data management and statistical analysis

The Data Collection Group manually entered the information obtained through paper questionnaires and face‐to‐face interviews into an online Google Form that was used for collecting data online. Subsequently, the data was extracted directly from the Google Form and transferred to an Excel spreadsheet. Finally, the raw data in the Excel sheet was transformed into a format that could be used with statistical software.

**Figure 1:**
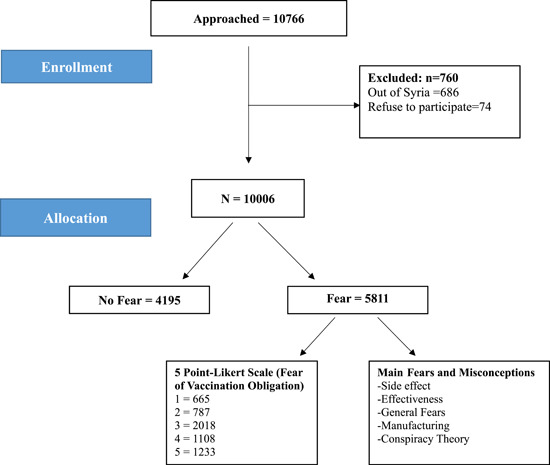
Shows the distribution of participants in the study.

**Figure 2:**
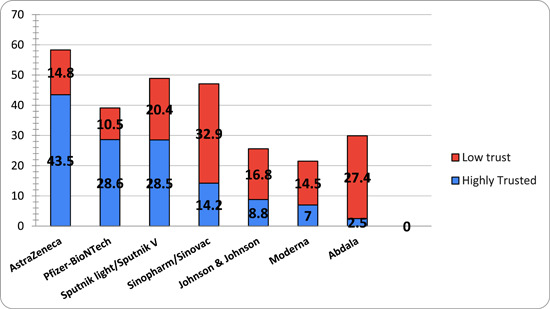
Shows the percentage of participants' trust in each vaccine.

All analyses were performed using SPSS Inc software version 26 on Microsoft Windows and reported as frequencies and percentages (for categorical variables). Analysis groups were defined as: gender, age, place of residence, education, field of study, and so forth. There were no missing data in the final data set used for analysis. A two‐sided *χ* ^2^ test was applied to assess relationships between categorical sociodemographic variables and responses to the question (Do you have fears about the COVID‐19 vaccine?). Figure 1 discusses how data was handled. Figure 2 and tables were used to display the main results. *p* < 0.05 were considered statistically significant.

## 3 RESULTS

Out of 10,766 participants, 10,006 have completed the survey. Table 1 shows the demographical characteristics of the study population which was divided into two groups based on the question “Do you have fears about the COVID‐19 vaccine?” Most of the study population are female (*n* = 6048; 60.4%), from the (18–24) age group (*n* = 5908; 59%), and university degree holders (*n* = 7304; 73%). Half of the participants belonged to the medical sector, and approximately half of them (49%) had concerns about the COVID‐19 vaccine. The Majority live in an urban setting (*n* = 8015; 80.1%), mainly in Aleppo, Hama, and Damascus. Half of the participants belonged to the medical sector, and approximately half of them (49%) had concerns about the COVID‐19 vaccine. Only 20.5% of respondents reported having health insurance, and this group had fewer concerns compared to those who did not have health insurance *χ* ^2^ (1, *N* = 10006) = 12.7, *p* < 0.001) (Table 1).

**Table 1:** Social and demographic characteristics of the participants^a^.

Variable		Total = 10006	Yes = 5811	No = 4195	*χ* ^2^	*p* Value
Gender						
	Male	3958 (39.6%)	2022 (34.8%)	1936 (46.2%)	131.3	<0.001
	Female	6048 (60.4%)	3789 (65.2%)	2259 (53.8%)		
Age						
	18–24	5908 (59%)	3537 (60.9%)	2371 (56.5%)	36.8	<0.001
	25–44	2922 (29.2%)	1678 (28.9%)	1244 (29.7%)		
	45–65	1003 (10%)	517 (8.9%)	486 (11.6%)		
	>65	173 (1.7%)	79 (1.4%)	94 (2.2%)		
Place of residence						
	City inside Syria	8015 (80.1%)	4692 (80.7%)	3323 (79.2%)	3.5	0.06
	Countryside in Syria	1991 (19.9%)	1119 (19.3%)	872 (20.8%)		
Governorate						
	Damascus	1816 (18.1%)	1016 (17.5%)	800 (19.1%)	134.1	<0.001
	Damascus countryside	647 (6.5%)	374 (6.4%)	273 (6.5%)		
	Aleppo	1909 (19.1%)	1303 (22.4%)	606 (14.4%)		
	Homs	1021 (10.2%)	553 (9.5%)	468 (11.2%)		
	Hama	1831 (18.3%)	961 (16.5%)	870 (20.7%)		
	Latakia	1165 (11.6%)	654 (11.3%)	511 (12.2%)		
	Tartous	947 (9.5%)	580 (10%)	367 (8.7%)		
	Eastern Governorate (Deir ez‐Zor, Raqqa, Alhasakah)	200 (2%)	166 (3.4%)	84 (2.0%)		
	Southern Governorate (Daraa, Al‐Suwayda, Quneitra)	390 (3.9%)	220 (2.0%)	170 (4.1%)		
	Idlib	80 (0.8%)	35 (0.6%)	45 (1.1%)		
Educational level						
	I did not go to school	163 (1.6%)	88 (1.5%)	75 (1.8%)	23.4	<0.001
	Finished middle school	568 (5.7%)	299 (5.1%)	269 (6.4%)		
	Passed high school	645 (6.4%)	350 (6%)	295 (7%)		
	University\Institute	7304 (73%)	4346 (74.8%)	2958 (70.5%)		
	Master\PhD	1326 (13.3%)	728 (12.5%)	598 (14.3%)		
Field of study						
	Medical sector	5021 (50.2%)	2856 (49.1%)	2165 (51.6%)	27.0	<0.001
	Other (nonmedical)	3851 (38.5%)	2350 (40.4%)	1501 (35.8%)		
	I did not study at university	1134 (11.3%)	605 (10.4%)	529 (12.6%)		
Health insurance						
	Yes	2053 (20.5%)	1121 (19.3%)	932 (22.2%)	12.7	<0.001
	No	7953 (79.5%)	4690 (80.7%)	3263 (77.8%)		
Is COVID‐19 a threat to the world?						
	No threat	444 (4.4%)	226 (3.9%)	218 (5.2%)	80.6	<0.001
	Small risk	3250 (32.5%)	2038 (35.1%)	1212 (28.9%)		
	High risk	5138 (51.3%)	2799 (48.2%)	2339 (55.8%)		
	I do not know	1174 (11.7%)	748 (12.9%)	426 (10.2%)		
Is COVID‐19 a threat to your personal life?						
	No threat	1447 (14.5%)	804 (13.8%)	643 (15.3%)	48.8	<0.001
	Small risk	4901 (49%)	2927 (50.5%)	1974 (47.1%)		
	High risk	2482 (24.8%)	1326 (22.8%)	1156 (27.6%)		
	I do not know	1176 (11.8%)	754 (13%)	422 (10.1%)		
Did you or anyone of your family members get infected COVID‐19?						
	Yes	5459 (54.6%)	3151 (54.2%)	2308 (55%)	46.7	<0.001
	No	1577 (15.8%)	815 (14%)	762 (18.2%)		
	Not sure but I had similar symptoms	2970 (29.7%)	1845 (31.8%)	1125 (26.8%)		
Was the COVID‐19 laboratory confirmed using a nasal swab (PCR test)?						
	Yes	1808 (18.1%)	1011 (17.4%)	797 (19%)	4.3	0.2
	No	7593 (75.9%)	4441 (76.4%)	3152 (75.1%)		
	Not sure	605 (6%)	359 (6.2%)	246 (5.9%)		
Has anyone in your family died of COVID‐19?						
	Yes	1426 (14.3%)	810 (13.9%)	616 (14.7%)	7.3	0.03
	No	8195 (81.9%)	4753 (81.8%)	3442 (82.1%)		
	Not sure	385 (3.8%)	248 (4.3%)	137 (3.3%)		
Do you think that the country of manufacture the vaccine affect the degree of confidence in the vaccine?						
	Yes	5464 (54.6%)	3354 (57.7%)	2110 (50.3%)	116.3	<0.001
	No	2637 (26.4%)	1297 (22.3%)	1340 (31.9%)		
	Not sure	1905 (19%)	1160 (20%)	745 (17.8%)		
Does the World Health Organization (WHO) approval of a vaccine license influence your decision to take it?						
	Yes	5537 (55.3%)	2840 (48.9%)	2697 (64.3%)	247.2	<0.001
	No	1977 (19.8%)	1363 (23.5%)	614 (14.6%)		
	Not sure	1255 (12.5%)	785 (13.5%)	470 (11.2%)		
	I do not know this organization	1237 (12.4%)	823 (14.2%)	414 (9.9%)		
Did you take the vaccine?						
	Yes, I took the vaccine in its full course with two doses	2445 (24.4%)	779 (13.4%)	1666 (39.7%)	1862.4	<0.001
	Yes, I only took the first dose	872 (8.7%)	423 (7.3%)	449 (10.7%)		
	Yes, I took the full course of the vaccine with a single dose	1080 (10.8%)	355 (6.1%)	725 (17.3%)		
	No	5511 (55.1%)	4235 (72.9%)	1276 (30.4%)		
	Yes, with a booster dose	98 (1%)	19 (0.3%)	79 (1.9%)		
Are you planning to take the vaccine in the future?						
	Yes	1412 (14.1%)	705 (12.1%)	707 (16.9%)	2341.4	<0.001
	No	1821 (18.2%)	1551 (26.7%)	270 (6.4%)		
	Still hesitant	2278 (22.8%)	1979 (34.1%)	299 (7.1%)		
What is/are the Source(s) of the main information related to the vaccine?						
	Social media	4125 (41.2%)	2373 (40.8%)	1752 (41.8%)	6.9	0.6
	Internet search engines (Google)	2864 (28.6%)	1645 (28.3%)	1219 (29.1%)		
	Relatives and friends	2439 (24.4%)	1404 (24.2%)	1035 (24.7%)		
	Publications of refereed scientific journals and societies	2229 (22.3%)	1327 (22.8%)	902 (21.5%)		
	Community and governmental education campaigns carried out by the Ministry of Health	2138 (21.4%)	1241 (21.4%)	897 (21.4%)		
	I am not looking for information on COVID‐19 vaccines	1863 (18.6%)	1099 (18.9%)	764 (18.2%)		
	My own doctor	1507 (15.1%)	897 (15.4%)	610 (14.5%)		
	TV and Radio	1259 (12.6%)	724 (12.5%)	535 (12.8%)		

a The table was divided based on the question “Do you have fears about the COVID‐19 vaccine?”

We recorded 5811 participants who still have fears and anxieties about the COVID‐19 vaccine, and our data proved that this group had a significant difference compared to the another group in the following results (Table 1): Females are more likely to have fears compared to men (*χ* ^2^ (1, *N* = 10006) = 131.25), (*p* < 0.001), 4346 (74.8%) hold university degree (*χ* ^2^ (4, *N* = 10006) = 23.4) (*p* < 0.001) and 3151 (54.2%) has a history of symptomatic COVID‐19 (*χ* ^2^ (2, *N* = 10006) = 46.7) (*p* < 0.001). It is worth noting that the PCR test was only performed in 1011 (17.4%) of this group which may indicate misdiagnoses of COVID‐19 in participants who did not perform a PCR test and yet thought their symptoms were related to COVID‐19 only. However the majority of this group 4753 (81.8%) did not lose any family members during the pandemic, 3354 (57.7%) believed that the manufacturing country of the vaccine affects their confidence in it (*χ* ^2^ (2, *N* = 10006) = 116.3) (*p* < 0.001), 2840 (48.9%) of the participants' choice of the vaccine depended on the approval of the World Health Organization (*χ* ^2^ (2, *N* = 10006) = 116.3) (*p* < 0.001), and they think that COVID‐19 poses a great danger to society and a small risk to themselves as individuals. Table 5 shows that 40.3% of people with fears about COVID vaccines tend to be concerned about the obligation of vaccines by the government. Although, 5811 participants have fears of vaccination, 1576 (27.1%) of them have vaccinated, while 1979 (34.1%) participants were still hesitant, and 1551 (26.7%) participants have refused to be vaccinated. In contrast, 1276 (30.4%) people did not have any fears about vaccines, and they were unvaccinated. Social media and internet search engines were the main sources of information for the majority of this group followed by relatives and friends, while more reliable sources of information such as scientific papers and governmental campaigns were not as common as social media.

Data from 10,006 participants were analyzed, approximately half (*N* = 5511, 55.1%) did not take the COVID‐19 vaccine and more than half of them (*n* = 4099) were hesitant or not willing to take the vaccine in the future. Figure 2 answers the following questions: Which vaccines do you trust? and Which vaccines do you have low confidence in? As a result, AstraZeneca is the most trusted vaccine (43.5%) compared to Sinopharm/Sinovac, which has the lowest confidence (32.9%) among our study population (Table 2).

**Table 2:** Shows the number of particpants based on their trust level of each vaccine.

Vaccine type	Percentage (%)	Percentage (%)
	Highly trust	Low trust
AstraZeneca	4354 (43.5)	1484 (14.8)
Pfizer‐BioNTech	2865 (28.6)	1050 (10.5)
Sputnik light/Sputnik V	2861 (28.5)	2045 (20.4)
Sinopharm/Sinovac	1416 (14.2)	3291 (32.9)
Johnson & Johnson	884 (8.8)	1683 (16.8)
Moderna	701 (7.0)	1452 (14.5)
Abdala	246 (2.5)	2744 (27.4)

We divided our population's fears into five subgroups (Table 3). The majority of fears belonged to the “Side Effects” subgroup as well the “Effectiveness.” The main concerns on COVID‐19 vaccines were the rapid development of vaccines (41.8%), fears of blood clots (39.5%), the fears of common side effects (36.9%), and allergic reactions (27.5%).

**Table 3:** Syrian's concerns about COVID‐19 vaccines.

*Side effects*	
Fear of blood clots	2297 (39.5)
Fear of common side effects (pain, swelling, or redness at the injection site, chills, feeling tired, headache, muscle, and joint aches	2146 (36.9)
Fear of severe allergic reaction	1598 (27.5)
One of my acquaintances contracted COVID‐19 after taking the vaccine	966 (16.6)
COVID‐19 vaccines will affect my fertility	746 (12.8)
COVID‐19 vaccines cause autoimmune disease	659 (11.3)
I have a chronic disease and I fear its exacerbation	487 (8.4)
COVID‐19 vaccines cause severe menstrual disorder	185 (3.2)
COVID‐19 vaccines cause paralysis	154 (2.7)
The vaccines are not safe for pregnant/breastfeeding	127 (2.2)
COVID‐19 vaccines cause retardation	110 (1.9)
*Effectiveness*	
COVID‐19 vaccines were developed too fast to be trust	2427 (41.8)
The vaccines are experimental	1138 (19.6)
Taking the vaccine causes Covid‐19 infection	610 (10.5)
*General fears*	
My doctor warning to take COVID‐19 vaccines	987 (17.0)
I do not trust the doctor and nurses in hospitals	806 (13.9)
Fear of needles	410 (7.1)
I can not afford the vaccine	107 (1.8)
*Manufacturing*	
The actual vaccine is not distributed in our country	1245 (21.4)
Fear of unknown substances used in the vaccine	371 (6.4)
*Conspiracy theory*	
COVID‐19 vaccine is a secret plan to reduce the population	787 (13.5)
COVID‐19 vaccines are used to alter the human DNA	493 (8.5)

It is worth noting that (20.8%) of the participants who belonged to the medical sector had major concerns about blood clotting (Table 4) even though the real number of such incidents is quite low. These results alongside the high prevalence of some misconceptions already stated above can be linked to the widespread misleading sources of information especially social media which is more accessible to the public and affect the general opinion on a large scale. Table 5 shows that the majority of people with fears about COVID‐19 vaccines tend to have mild to extreme concerns about the obligation of vaccination. However, less than 25% chose points 1 or 2 on the Likert scale.

**Table 4:** Main fears according to field of study.

Field of study in university	Fears	*N* (%)
Medical sector		
	Rapidly developed vaccines	1335 (23.0%)
	Fear of blood clots	1207 (20.8%)
	Fear of common side effects (pain, swelling, or redness at the injection site, chills, feeling tired, headache, muscle, and joint aches)	1059 (18.2%)
	Fear of allergic reaction	801 (13.8%)
Other (nonmedical sector)	Rapidly developed vaccines	929 (15.9%)
	Fear of blood clots	878 (15.1%)
	Fear of common side effects (pain, swelling, or redness at the injection site, chills, feeling tired, headache, muscle and joint aches	866 (14.9%)
	Fear of allergic reaction	654 (11.3%)
I did not study at university	Rapidly developed vaccines	163 (2.8%)
	Fear of common side effects (pain, swelling, or redness at the injection site, chills, feeling tired, headache, muscle and joint aches	221 (3.8%)
	Fear of blood clots	212 (3.6%)

**Table 5:** Fears of vaccination obligation.

5 point‐Likert scale	Frequency	%
1	665	11.4
2	787	13.5
3	2018	34.8
4	1108	19.1
5	1233	21.2
Total	5811	

## 4 DISCUSSION

The study revealed that more than half of the population surveyed expressed hesitancy towards receiving the COVID‐19 vaccine, with reasons behind vaccine hesitancy classified into five groups: efficacy, fears of side effects, general concerns, manufacturing‐related fears, and conspiracy beliefs. Specifically, the rapid development of the vaccines and formation of blood clots were the most prominent concerns about vaccines. Furthermore, females, younger age groups, health‐care professionals, and individuals with higher levels of academic education showed lower rates of vaccine acceptance. In terms of vaccine trust, AstraZeneca was the most trusted while Sinopharm/Sinovac had the lowest confidence rate. Interestingly, a previous COVID‐19 infection in a family member led to increased vaccine hesitancy. The findings suggest the need for targeted communication strategies to address specific concerns of different demographic groups and improve vaccine uptake.

The aim of this study was to investigate the reasons behind vaccine hesitancy among Syrians. Of the 10,006 participants, approximately half (*n* = 5511, 50.1%) did not take the COVID‐19 vaccine, and more than half of them were hesitant or not willing to take the vaccine in the future. This low rate of vaccine acceptance can be attributed to pre‐existing fears related to the COVID‐19 vaccine, which were reported in 58.07% of participants. This is consistent with a study of Arabs in and outside the Arab region,^13^ but contrasts with countries like China and the United States which have shown high levels of vaccine acceptance.^14^, ^15^ Differences in acceptance rates and patterns can be ascribed to the different demographic and socioeconomic characteristics of these populations.

The five main reasons identified for vaccine hesitancy in Syria were fear of efficacy, followed by concerns about side effects, general concerns, manufacturing‐related reasons and conspiracy beliefs. The most feared side effect was the development of blood clots, which may be related to the use of the AstraZeneca vaccine in Syria, as thromboembolic events have been reported following this vaccine.^16^ Concerns about efficacy were related to the short period of time within which COVID‐19 vaccines were developed, which has raised concerns among the public regarding their efficacy and safety.^17^

Comparable results have been reported in studies of different populations, including in Saudi Arabia, where fear of side effects, efficacy concerns, and conspiracy beliefs were identified as barriers to acceptance of COVID‐19 vaccination.^18^ Similar findings were reported in an international survey among low and middle‐income countries, which showed that fear of efficacy was the most common reason for vaccine hesitancy, followed by concerns about side effects and conspiracy beliefs, respectively.^19^ According the to the previous studies, 79.9% of the population in Saudi Arabia feared the side effects that could result from the COVID‐19 vaccines, alongside 74.1% in Malaysia, 67.4% in Bangladesh, and 59.2% in Thailand. While our study had further elaborated on the feared side effects; showing that blood clots, common side effects such as pain, headaches or fever and allergic reactions were behind vaccine hesitancy in Syria. Similarly, regarding efficacy concerns, 44.5% of the population in Thailand believed that COVID‐19 vaccines are not effective, compared to 36.4% in Mali, and 23.4% in Saudi Arabia. Interestingly, almost half of the population in Syria thought that COVID‐19 vaccines were not effective given that they did not have enough times to be experimented and properly developed to be considered safe; thereby increasing people's hesitancy toward vaccination.

This study identified several factors associated with vaccination hesitancy and fears among Syrians. The female gender has been significantly associated with more fears regarding the COVID‐19 vaccine. The literature on gender and COVID‐19 vaccine acceptance has yielded varied results, with most studies indicating that females were less likely to accept the vaccine,^19^, ^20^ whereas a study conducted in 19 countries reported slightly higher acceptance rates in females.^21^ We found a significant difference between fears about the COVID‐19 vaccine and the different age groups, low rates of vaccine acceptance were observed in young age groups (18–24). This can be a result of the uneven distribution of people of different age groups within our sample and the misleading and wrongful information younger people receive online, being the most active on social media platforms, which is the main source of information for almost half of our participants. This highlights the need to address the widespread misconceptions and false information that could adversely affect people's acceptance of vaccination. A number of studies have reported similar results,^19^, ^20^ contrary to a study conducted in Jordan reporting higher levels of vaccine acceptance in younger people, which was attributed to the mostly young population of Jordan, and the lack of accessibility to the elderly through online‐based surveys.^22^

The current study showed similar rates of vaccine hesitancy among people living in the cities and countryside of Syria, contrary to common belief. However, there has been a statistical significance between the origin governorate of the participants and their COVID‐19 vaccines‐related fears. This suggests the need for governorate‐specific solutions to increase vaccine acceptance. We also found a significant correlation between fears of COVID‐19 vaccines and the level of academic education, with higher rates of fears and concerns reported in people of higher level of education, which is contrary to many studies reporting lower level of vaccine acceptance among persons with lower educational level.^13^, ^19^ This may be due to the under‐representation of people with lower level of academic education, and the tendency of participants with high level of education to do more research and question the efficacy and long term side effects of receiving newly emerging vaccines, which was actually demonstrated in a number of studies, including ours, that the most common reason of vaccine hesitancy is the fear of side effects.^18^, ^19^

The field of study has also significantly affected the rates of fears of COVID‐19 vaccines among our population. Previous studies indicated that healthcare workers were more willing to accept the COVID‐19 vaccine,^23^ whereas our study showed high rates of hesitancy in people in the medical sectors. This raises a red flag given that healthcare worker is at high risk of COVID‐19 infection being at direct contact with COVID‐19 patients and they form a trusted source of COVID‐19 and vaccine‐related awareness and are able to influence millions of people, which calls for further investigation of the underlying reasons behind these fears and apply the suitable solution to increase vaccination rates and achieve a stronger network of healthcare workers.

We also found a significant correlation between the fear of vaccination and having a family member who was infected with or died of COVID‐19, with the majority of these individuals being hesitant to take the vaccine. Conversely, it has been reported that infection of COVID‐19 in a family member or close social network may increase people's willingness to get vaccinated. However, the impact of the number of cases and the history of COVID‐19 infection on vaccine acceptance remains inconclusive.^24^

Although the majority of participants who refused vaccination had fears and concerns regarding the COVID‐19 vaccines, 30% of them did not fear the vaccination. This may suggest deeper issues regarding the culture of immunization and vaccination in general, as well as a lack of proper awareness of the severity of the pandemic overall. Future studies could explore these deeper issues and create strategies to address vaccine hesitancy, such as increasing awareness campaigns and improving access to credible information and knowledge resources. Additionally, studies could examine the impact of vaccine mandates and policies on vaccine acceptance, particularly in populations with lower income and less access to healthcare resources.

The endorsement of COVID‐19 vaccines by the World Health Organization (WHO) influenced the participants' decision to receive the vaccine, which indicates that the WHO is a trusted source of information and more research published by the WHO can increase the rates of vaccination around the world.

Another factor that has proven to significantly affect acceptance rates is the vaccine's country of origin, with the majority of the population trusting the AstraZeneca vaccine of a British origin the most, followed by Pfizer‐BioNTech of a US origin and Sputnik light/Sputnik V of a Russian origin; This is consistent with some studies but contrasts with others that have reported higher levels of trust in vaccines of other origins. For example, a study of the Polish population reported the highest level of trust in the CureVac (CVnCoV) vaccine, followed by the Johnson & Johnson (Ad26.COV2.S) manufactured in Germany and the United States, respectively.^25^

Another potential reason for irrational fears of COVID‐19 vaccination is the mental health status of the participants and the psychological effect of the COVID‐19 pandemic, which has had a particularly devastating impact on the Syrian population due to years of war and conflict. A previous study on the Syrian population has shown that 44% of them had severe mental disorder, 36.9% had full PTSD symptoms and 27% had both severe mental disorder and full PTSD symptoms.^26^ It was reported that mental health disorders negatively affected vaccine hesitancy,^27^ which could be the case in our study. Further research is required to study the mental health‐specific effects on vaccine acceptance.

Given that fear of side effects was reported the most common reason for vaccine hesitancy, it is important to provide appropriate awareness regarding the types of vaccines, the targeted age groups, short‐ and long‐term effects and many other related information that would help promote the importance and efficacy of COVID‐19 vaccines. This is expected to increase vaccine acceptance and vaccination rates, particularly among females, who have expressed fear of effects on menstruation, childbearing, breastfeeding, fertility, and so forth. Addressing remaining fears, efficacy concerns of the COVID‐19 vaccines have been reported as the second most common fear towards vaccination, with most people fearing that the vaccine was developed too fast and that there has not been enough time to test vaccine safety and efficacy or that taking the vaccine might cause infection with COVID‐19.

Conspiracy beliefs have been demonstrated worldwide. Our study showed relatively large proportion of the population who had conspiracy beliefs that the COVID‐19 vaccines are used to alter the human DNA, and that COVID‐19 pandemic/vaccines being a plot for depopulation, these beliefs have been reported in a number of studies among different populations that believed in such conspiracy theories and had similar fears.^13^, ^19^, ^22^ This creates an international responsibility to correct misinformation and debunk conspiracy beliefs and make evidence‐based information more accessible for the general population to increase the level of societies awareness and achieve higher rates of vaccination.

To address vaccine‐related concerns and improve vaccine uptake among the Syrian population, several public health strategies could be implemented based on the findings of this study. First, targeted educational campaigns should be developed to disseminate accurate information about the vaccine through social media platforms and other online platforms, as these were found to be the main sources of information for the majority of participants. Second, healthcare providers should be engaged and their concerns addressed, which could help improve vaccine uptake among healthcare workers, who can then serve as advocates for the vaccine among their patients and the community. Third, misconceptions about the vaccine should be addressed by providing accurate information and dispelling myths about its safety and efficacy. Fourth, transparency in the vaccine approval process should be ensured to build trust in the vaccine, which was found to be affected by the manufacturing country. Finally, concerns about side effects should be addressed by providing information about the common side effects of the vaccine and reassuring the public that these side effects are usually mild and short‐lived. These strategies should be implemented through policy briefs, guidelines, and educational campaigns to improve vaccine uptake and address vaccine‐related concerns in Syria.

Several limitations arose in this study, such as the fact that the study was conducted at a certain point in time, when the fourth wave of the COVID‐19 pandemic peaked in February with the Omicron variant confirmed. This may have affected the population's attitude towards COVID‐19 vaccination, making them more hesitant to receive the vaccine due to fear of infection and severe symptoms or even death.

Moreover, the situation in Syria is particularly challenging, as the country has been subjected to a range of economic sanctions and restrictions in recent years. These sanctions can have a significant impact on the behavior of populations. When people are experiencing economic difficulties, they may be more hesitant to seek medical care, including vaccinations. As a result, it is crucial for policymakers and public health officials to carefully consider the economic context in which vaccination campaigns are taking place and to address any economic barriers that may be preventing people from accessing vaccines.

The findings of this study are based on Syrian population so the findings may not be generalizable to other populations with different cultural, social, economic, or political backgrounds. Therefore, caution should be taken when interpreting the findings and applying them to other populations or settings. It is recommended that future studies should aim to replicate and extend the findings to other populations and settings to increase the external generalizability of the results.

Nonetheless, the findings of this study provide valuable insights into the attitudes of Syrians towards emerging vaccines, and addressing vaccine hesitancy and related fears should be considered in future action plans to increase positive public response and vaccination rates. This will reduce morbidity and mortality from vaccine‐preventable diseases and enable adequate public health policies.

## 5 CONCLUSION

The most common reasons for refusing the COVID‐19 vaccine in Syria belonged to vaccines' side effects, followed by concerns about efficacy, general worries, manufacturing‐related issues, and conspiracy beliefs. However, the COVID‐19 vaccine is considered the most promising measure for controlling the spread of infection; its success will depend on the global acceptance. High variability in vaccine acceptance hesitancy can hinder the efforts to end the COVID‐19 pandemic, so, addressing the barriers to acceptance is crucial for achieving maximum vaccination coverage. It is important to consider these factors when interpreting the results of any study on vaccine attitudes among the Syrian population.

## AUTHOR CONTRIBUTIONS

**Mohamad Klib**: Conceptualization; data curation; investigation; methodology; project administration; supervision; visualization; writing—original draft; writing—review & editing. **Munir Ghandour**: Conceptualization; methodology; writing—original draft; writing—review & editing. **Osama Alazki**: Conceptualization; data curation; methodology; writing—original draft; writing—review & editing. **Ayman I. Nabhan**: Conceptualization; methodology; project administration; supervision; writing—review & editing. **Fatima A. Idres**: Writing—original draft. **Homam Alolabi**: Formal analysis. **Majd S. Khaddour**: Conceptualization; methodology; project administration; supervision; writing—original draft. **Jaafar Zahlout**: Conceptualization; methodology; validation. **Farah Albakkar**: Writing—original draft. **Hasan M. M. Hamoud**: Writing—original draft. **Hasan N. Al Houri**: Writing—original draft. **Bana Z. Alafandi**: Methodology; writing—original draft; writing—review & editing. **Data Collection Group**: Resources.

## DATA COLLECTION GROUP

List names of collaborators: Hassan Karahbatak, Heba Cheikh Othman, Mohamad Abdalrahman Alayoubi, Mohammad Ekbal Atik, Mohammad Ghieh, Mohammad Rabee. Mslmani, Zain Houssen Hasan, Zienab Klib (Faculty of Medicine, Damascus University). Okbah Mohamad (Plastic and reconstructive surgery, Damascus University). Batoul Mohammad (Faculty of Pharmacy, Damascus University). Mhd Maher Marzouk, Mohamad Wassim Almasri, Nagham Zaino (Faculty of Dentistry, Damascus University). Eman Awwad, Ghada Bawadekji, Maria Chakhide, Jude Sumakie, Mohamed Amin Akil, Nour Alsamou Alnajjar, Ola Ramadan, Qamar Alamki, Rahaf Maeri, Rama Zammar, Salma Mahmoud Omar, Sana Zammar, Souzan Abboud (Faculty of Medicine, University of Aleppo). Alaa Khaddour, Hazar Husien, Iyad Ali, Maha Mansour, Mary Hassoun, Rita Mohammad, Zeyazan Ebrahim (Faculty of Medicine, Tishreen University). Ghina Ismail (Department of Gastroenterology, Faculty of Medicine, Tishreen University). Agharid Sejaa, Ahmad Yasin Saleh, Elias Afesa (Faculty of Medicine, Al‐Andalus University for Medical Sciences). Zeina Al‐khatib (Faculty of Pharmacy, Al‐Andalus University for Medical Sciences). Aya Younes, Mohamad Mostafa, Mustafa Youssef (Faculty of Medicine, Tartous University). Agead Sheikh Sobeh, Ahmad Yaman Baker, Ahmed Sabbagh, Alaa Koujah, Bahaa Almalohi, Hadi Alabdullah, Hasan Khaldoun Haydar, Kenana Tawashi, Mostafa Ghandour (Faculty of Medicine, Hama University). Abdulrahman Khaldoun Haydar, Sumiah Ghandour (Faculty of Dentistry, Arab private University for Science and Technology). Ruba Almenchaf, Dana Alsibai, Nour Atmaz Alsibai (Faculty of Medicine, Al‐Baath University). Sumaya Alsabea (Faculty of Pharmacy, Al‐Wataniya Private University). Roaa Musa (Al‐Sham Private University), Lina Aljaber (Faculty of Medicine, Al‐Furat University).

## CONFLICT OF INTEREST STATEMENT

The authors declare no conflict of interest.

## ETHICS STATEMENT

The study was approved by the University of Aleppo, Faculty of Medicine, and performed as per Helsinki Declaration principles. Ethical approval was obtained from the Institutional Review Board (IRB), Faculty of Medicine, University of Aleppo and the study was performed as per Helsinki Declaration principles (No:569). All authors have read and approved the final version of the manuscript corresponding author had full access to all of the data in this study and takes complete responsibility for the integrity of the data and the accuracy of the data analysis.

## TRANSPARENCY STATEMENT

The lead author Mohamad Klib affirms that this manuscript is an honest, accurate, and transparent account of the study being reported; that no important aspects of the study have been omitted; and that any discrepancies from the study as planned (and, if relevant, registered) have been explained.

## Data Availability

The authors confirm that the data supporting the findings of this study are available within the article to the editor. All relevant data and materials are included in the manuscript. Any additional information can be obtained from the corresponding author upon reasonable request.
